# Changes in the Total Fecal Bacterial Population in Individual Horses Maintained on a Restricted Diet Over 6 Weeks

**DOI:** 10.3389/fmicb.2017.01502

**Published:** 2017-08-11

**Authors:** Kirsty Dougal, Patricia A. Harris, Susan E. Girdwood, Christopher J. Creevey, Gemma C. Curtis, Clare F. Barfoot, Caroline M. Argo, Charles J. Newbold

**Affiliations:** ^1^Institute of Biological Environmental and Rural Sciences, Aberystwyth University Aberystwyth, United Kingdom; ^2^Equine Studies Group, WALTHAM Centre for Pet Nutrition Melton Mowbray, United Kingdom; ^3^Department of Obesity and Endocrinology, Faculty of Health and Life Sciences, University of Liverpool Neston, United Kingdom; ^4^MARS Horsecare UK Ltd. Old Wolverton, United Kingdom; ^5^School of Veterinary Medicine, Faculty of Health and Medical Sciences, University of Surrey Guildford, United Kingdom

**Keywords:** equine, microbiome, stability, hindgut, core

## Abstract

Twelve mature (aged 5–16 years) horses and ponies of mixed breed and type were fed restricted (1.25% BM Dry matter) quantities of one of two fiber based diets formulated to be iso-caloric. Diet 1 comprised of 0.8% body mass (BM) of chaff based complete feed plus 0.45% BM low energy grass hay (the same hay used for both diets). Diet 2 comprised 0.1% BM of a nutrient balancer plus 1.15% BM grass hay. Fecal samples were collected at week 10 and week 16. DNA was extracted and the V1-V2 regions of 16SrDNA were 454-pyrosequenced to investigate the bacterial microbiome of the horse. The two most abundant phyla found in both diets and sampling periods were the *Firmicutes* and *Bacteroidetes.* There was a clear reduction in *Bacteroidetes* with a concordant increase in *Firmicutes* over time. There was a limited degree of stability within the bacterial community of the hindgut of horses, with 65% of bacteria retained, over a 6 week period whilst on a uniform diet. The presence of a core community defined by being present in all samples (each animal/diet combination) included in the study and being present at 0.1% relative abundance (or greater) was identified. In total 65 operational taxonomic units (OTUs) were identified that fit the definition of core making up 21–28% of the total sequences recovered. As with total population the most abundant phyla were the *Bacteroidetes* followed by the *Firmicutes*, however there was no obvious shift in phyla due to period. Indeed, when the relative abundance of OTUs was examined across diets and periods there was no significant effect of diet or period alone or in combination on the relative abundance of the core OTUs.

## Introduction

Investigations into the bacterial community in the large intestine of the horse have thus far mainly involved samples from single time points. Work which has explored samples over time has focused on laminitis progression (Milinovich et al., 2007, 2008), or changes in diet (de Fombelle et al., 2001; Drogoul et al., 2001; Julliand et al., 2001; Respondek et al., 2007; Müller et al., 2008). To our knowledge only two studies have investigated the temporal stability of the bacterial community in the equine hindgut whilst on a uniform diet, using culture independent methods (Willing et al., 2009; Blackmore et al., 2013). Willing et al. (2009) identified greater stability associated with a high fiber compared to a concentrate supplemented diet. Blackmore et al. (2013) found the bacterial community to be significantly different in structure after 10 weeks on a uniform fiber based diet. These two studies also confirmed the findings, from single time point data, that there is large inter-animal variability (Costa et al., 2012; Steelman et al., 2012).

In humans, the gut bacterial population has been shown to be relatively stable over time in an adult individual regardless of diet. Stability of the structure of the bacterial community has been shown over 3 months (Costello et al., 2009), 6 months (Zoetendal et al., 1998), 1 year (Ley et al., 2006), and 5 years (Faith et al., 2013). Individuals show less change in their gut microbial population over 24 h than over 3 months but still retain significant similarity to the community structure at the initial time point (Costello et al., 2009). Regardless of diet, after 1 year 70% of bacterial strains have been shown to be retained in an individual adult with relatively little change over a subsequent 4 year period with the phyla *Bacteroidetes* and *Actinobacteria* showing greater stability than *Firmicutes* or *Proteobacteria* (Faith et al., 2013). Work in other species has also shown stability of the bacterial community over time; over 1 year in mice (Schloss et al., 2012), 8 years in chimpanzee (Degnan et al., 2012), and over 3 years in adult cheetahs (Becker et al., 2015)

We have previously shown that horses shared a relatively small core in terms of bacteria present in all animals fed the same diet (Dougal et al., 2014). It is not known if the change in community over time, demonstrated by Blackmore et al. (2013), reflects changes in the core bacterial community shared between animals or variability within the individual animal’s host specific profile. Dependent on where these changes occur, comment can be made on true stability of the bacterial community over time in the horse. We suggest that if the core bacterial community, shared in all animals at all times, remains relatively stable in terms of relative abundance over time, this may be indicative of a stable community which may have a degree of resilience to normal changes in the animals’ environment and management. Once the degree of temporal stability is known it will facilitate a greater understanding of the importance of the hindgut bacterial population in metabolic disease and dietary intervention. Here we present an investigation as to how stable the bacterial community of horse feces is over time by trying to identify a core population common to all animals and sampling points using 454 pyrosequencing.

## Materials and Methods

### Animal Trial and Sample Collection

Samples were obtained as part of a research trial undertaken at the University of Liverpool veterinary department (Argo et al., 2012). Twelve mature (aged 5–16 years) horses and ponies of mixed breed and type were recruited and loaned from clients of the university equine veterinary practice who sought assistance with weight correction for their animals. All were selected as being overweight or obese judged by a high body condition score (BCS) according to the method of Henneke et al. (1983) (mean BCS 7.8 ±0.18). All animals were deemed to have no dental abnormalities and were subject to anthelmintic treatment prior to starting on trial (see supporting information Supplementary Table S1 for metadata relating to the animals). The purpose of the primary investigation was to investigate weight loss in horses and ponies. As such, the horses and ponies were randomly assigned to two groups; each fed a different diet at 1.25% as dry matter (DM) of actual body mass (BM). The two diets were both fiber based and were formulated to be iso-caloric. Diet 1 comprised of 0.8% BM of a chaff based complete feed plus 0.45% BM low energy grass hay (the same hay used for both diets). Diet 2 comprised 0.1% BM of a nutrient balancer plus 1.15% BM grass hay (Supplementary Table S2 describes the nutrient composition of the chaff based feed, nutrient balancer and hay used to formulate the two diets, analysis carried out via the University of Liverpool). During the trial, all animals were housed in individual 5 m× 6m loose boxes and allowed paddock based exercise for 30 min daily, where possible, whilst wearing a muzzle to restrict grazing. All procedures were conducted in accordance with Home Office requirements, approved by the University of Liverpool’s Ethical Review Board and informed consent was obtained from all owners. The trial was run for 16 weeks and samples were obtained for microbiological investigation, for the purpose of this study, after 10 weeks then again after a further 6 weeks (giving two distinct sampling periods). These sampling time points were chosen so that the horses were on the same diet for 10 weeks to acclimatize, but at a time point when weight loss was predicted to cease. At week 10 animals had lost on average 4% of body weight, and by the second sample collection at week 16 had only lost a further 1%, showing body weight was reasonably stable during this sampling period. Two or three separate fecal samples were collected from each animal during sample day 1 and again during sample day 2. Freshly voided feces were selected and sub-sampled (approximately 500 g) from the central portion to minimize contamination by bedding and flooring. After collection samples were stored on ice until frozen at –80°C prior to freeze drying.

### DNA Extraction

Prior to extraction of nucleic acids, freeze dried samples were disrupted by bead beating. Freeze- dried samples (100 mg) were added to a 2 ml screw top tube and one autoclaved glass ball was added (4 mm, undrilled, G/0300/53, Fisher Scientific, United Kingdom). Samples were beaten for 90 s at 5000 rpm (maximum speed) in a Mini-Beadbeater^TM^ (Biospec products Inc., Bartlesville, OK, United States). DNA was then extracted using QIAGEN QIAamp^®^ DNA stool mini kits (Qiagen Ltd., United Kingdom) using the method described by Skrivanová et al. (2010).

### PCR Amplification of 16S rDNA

Amplification of the V1–V2 hyper-variable regions of 16S rRNA was carried out with primers 27F and 357R (Liu et al., 2007). The forward primer (5′-AGAGTTTGATCMTGGCTCAG-3′) carried the 454 Lib-L adaptor sequence B (5′-CCTATCCCCTGTGTGCCTTGGCAGTCTCAG-3′) and the reverse primer (5′-ACGAGTGCGTCTGCTGCCTYCCGTA-3′) carried the 454 Lib-L adaptor sequence A (5′-CCATCTCATCCCTGCGTGTCTCCGACTCAG-3′) followed by a 10 nucleotide sample specific barcode sequence. For each sample replicate PCR was performed in duplicate; a 25 μl reaction was prepared containing 5U μl^-1^ FastStart High Fidelity Enzyme Blend, 10× FastStart High Fidelity Buffer with 18 mM MgCl_2_ (Roche Diagnostics Ltd., Burgess Hill, United Kingdom), 0.2 mM of each dNTP (Promega UK Ltd. Southampton, United Kingdom) with each primer used at 0.2 μM. For each reaction 1 μl DNA template at 2.5–125 ng/μl (as per Roche FastStart high Fidelity system recommendations) was used. The conditions used were a hot start of 95°C for 10 min, 95°C for 2 min followed by 22 cycles of 95°C for 30 s, 60°C for 30 s, and 72°C for 45 s with a final extension at 72°C for 7 min. Reactions were amplified in a T100^TM^ thermal cycler (Bio-Rad, Hemel Hempstead, United Kingdom). Resultant amplicons were visualized on a 1% (w/v) TAE agarose gel to assess quality of amplification before pooling the duplicate reactions.

### Short Fragment Removal and Pooling of Libraries and Sequencing

Pooled PCR reaction products for all sample replicates were purified as per Roche technical bulletin 2011-007 (January 2012) ‘Short Fragment Removal Procedure for the Amplicon Library Preparation Procedure’ using Agencourt AMpure XP beads (Beckman Coulter Inc., Fullerton, United States). DNA concentration of the purified PCR products was assessed using an Epoch Microplate Spectrophotometer with a Take3 Micro-Volume plate (BioTek UK, Potton, United Kingdom) to enable equi-molar pooling of samples into four libraries each containing 36 to 39 samples with unique barcode sequences. Each library was further purified using the E-Gel^®^ System with E-Gel^®^ SizeSelect^TM^ 2% Agarose gel (Life Technologies Ltd, Paisley, United Kingdom). A final purification step using Agencout AMpure XP beads standard PCR purification procedure (Beckman Coulter Inc., Fullerton, United States) was carried out for each library. To assess purity of the sample libraries a quality control PCR was carried out for each as detailed in Roche technical bulletin 2011-007. All 25 μl reactions were prepared containing: 5U μl^-1^ FastStart High Fidelity Enzyme Blend, 10× FastStart High Fidelity Buffer with 18 mM MgCl_2_ (Roche Diagnostics Ltd., Burgess Hill, United Kingdom), 0.2 mM of each dNTP (Promega UK Ltd. Southampton, United Kingdom) with each primer used at 0.2 μM. Primers used were the same as the Lib-L adapter sequences (described previously) as recommended in the Roche Technical Bulletin 2011-007. For each reaction 1 μl of each library containing 2 × 10^8^ molecules/μl was used. The conditions used were 94°C for 11 min followed by 20 cycles of 94°C for 1 min, 60°C for 1 min and 72°C for 1 min with a final extension at 72°C for 10 min. On completion PCR products were incubated for 30 min at 37 C with 0.5 μl of Exonuclease I (New England BioLabs Ltd. Hitchin, United Kingdom). Reactions were amplified in a T100^TM^ thermal cycler (Bio-Rad, Hemel Hempstead, United Kingdom). Products from the quality control PCR were assessed for quality and purified libraries were quantified on an Agilent 2100 Bioanalyzer with a High Sensitivity DNA chip (Agilent Technologies UK Ltd, Stockport, United Kingdom). The sample libraries were subsequently sequenced using the Roche 454 GS FLX Titanium series sequencer following ‘emPCR Method Manual-Lib-L’.

### Sequence Filtering, Processing, and Statistical Analysis

Following sequencing data were combined and sample identification assigned to multiplexed reads using the MOTHUR software environment (Schloss et al., 2009). Data were de-noised by removing low quality sequences, sequencing errors and chimeras (quality parameters: maximum 10 homo-polymers, Q15 average over a 50 bp window, no mismatches allowed with barcode and 1 maximum with primer; Chimera check, both de novo and database driven using Uchime). Then sequences were clustered into operational taxonomic units (OTUs) at 97% identity using the CD-HIT-OTU pipeline^1^ (Li et al., 2012). OTUs containing fewer than four reads per individual diet/animal combination were excluded due to the likelihood of them being a sequencing artifact. Samples were normalized by randomly resampling the sequences used to the lowest number of sequences per sample (each diet/animal combination) using Daisychopper^2^. Taxonomic classification of OTUs was carried out using the Ribosomal Database Project (RDP) Classifier (Wang et al., 2007).

Data were prepared and tables and figures produced using Microsoft Excel and the ‘R’ software environment^3^ (version 2.15). Simpson and Shannon-Wiener diversity indices were calculated using normalized data as recommended to reduce over-inflation of true diversity in pyrosequencing data sets (Gihring et al., 2012). Species richness and diversity were then analyzed by two-way ANOVA using GenStat^®^ 12th edition, unless otherwise mentioned below *P*-values were considered significant <0.05.

The core community at OTU level in feces was defined by being present in all samples (each animal/diet combination) included in the study and being present at 0.1% relative abundance (or greater). The relative abundance of core OTUs between diets and periods was analyzed by two-way ANOVA using GenStat^®^ 12th edition using a Benjamini-Hochberg correction to account for multiple comparisons (Benjamini and Hochberg, 1995) and considered significant when the adjusted *p*-value was less than 0.1. Stability was measured as the mean abundance of OTUs shared between time periods 1 and 2. This was calculated for the total bacterial community at three different levels of relative abundance (0.01, 0.05, and 0.1%). The Jaccard index, a coefficient of community similarity, was calculated for each animal between the two time periods using the Vegdist function from the Vegan community ecology package in R.

To test for statistical differences in abundance of individual bacteria over time or between diets, data were firstly grouped by bacterial genus. Statistical analyses of differential abundances were carried out using the bioconductor package DESEQ2^1^ in the statistical package R. This parametric approach is designed for high-throughput sequencing count data and models the distribution of read counts from each OTU using a negative binomial distribution (Love et al., 2014). The design of the experiment used to analyze the data included two factors, time and diet and a term for their interaction. All resulting *p*-values were adjusted for multiple testing using the Benjamini and Hochberg (BH)^3^ approach (Benjamini and Hochberg, 1995) and considered significant when the adjusted *p*-value was less than 0.1.

### Nucleotide Sequence Accession Numbers

16S rRNA sequences were deposited with the EBI Sequence Read Archive (SRA) under study accession number: PRJEB20876.

## Results

The cessation of weight loss by week 10 (where the first sample was collected) was retrospectively confirmed (see Argo et al., 2012).

### Coverage and Diversity

In total 1,688,925 sequences of average length 358bp were obtained from 454 FLX Titanium sequencing of 24 samples. Quality filtering resulted in 599,160 high quality sequences. Sequences were clustered into 1868 unique OTUs across the complete data set. A phylogenetic tree was constructed (using the ‘hclust’ package in R with Bray Curtis dissimilarity) (Supplementary Figure S1), which indicated that all samples from an animal, on a sampling day (two or three across the day) clustered tightly together which allowed data from these samples to be pooled. This provided 13,259 sequences per animal, per sample day after normalization. Rarefaction curves (Supplementary Figure S2) showed that no sample curve had plateaued; indicating that complete sampling of these environments had not yet been achieved. Good’s coverage estimates, however, indicated that a large part of the diversity in all samples had been captured with the average sample coverage being 99.3% (*SD* = 0.3). Bacterial species diversity was significantly higher with diet 2 (Hay only) than diet 1 (Hay plus commercially prepared chaff based complete feed); Shannon–Wiener index; *p* = 0.04, there was no difference in diversity or richness between sample periods (**Table 1**).

**Table 1:** Diversity and richness of the bacterial communities in feces of horses over time (period 1 v period 2, 6 weeks apart) and diet (Diet 1 = hay plus chaff diet, Diet 2 = hay plus balancer).

	Period 1		Period 2		SED		
	Diet 1	Diet 2	Diet 1	Diet 2	Period	Diet	Period^∗^ diet
Species richness	880	905	779	928	31.6	31.6	44.7
Simpson’s diversity	0.985	0.986	0.986	0.990	0.001	0.002	0.003
Shannon–Wiener diversity	5.416	5.479	5.260	5.651	0.07	0.06^∗^	0.10^∗^

*SED, standard error of the difference. ^∗^*p* < 0.05*.

### Bacterial Phyla and Phylogenetic Relationships

**FIGURE 1:**
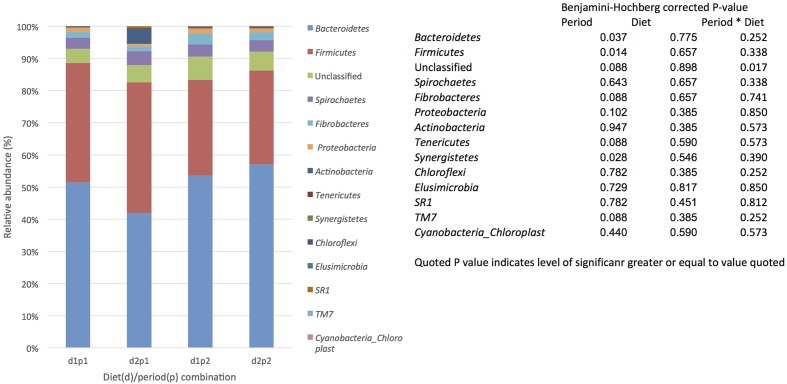
The effect of time (p: period 1 v period 2,6 weeks apart) and diet (d: diet 1 = hay plus chaff diet, diet 2 = hay plus balancer) on the relative abundance (%) of bacterial phyla in equine feces.

**FIGURE 2:**
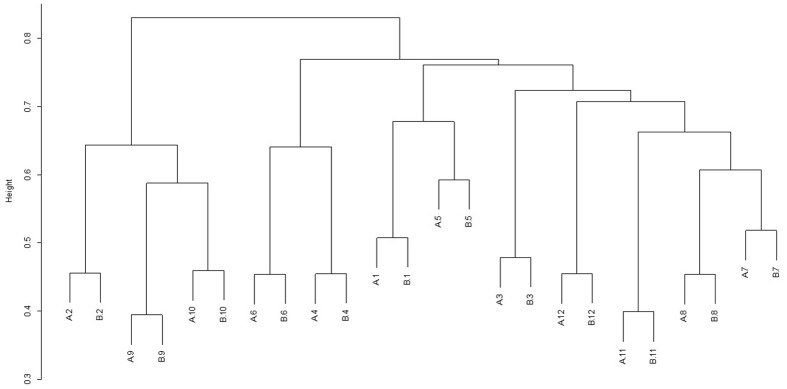
Dendrogram showing the clustering of samples from individual animals (1–12) during sample period 1 **(A)** and sample period 2 **(B)**. Animals 1–6 were on diet 1(hay plus chaff diet) and animals 7–12 on diet 2 (hay plus balancer diet).

For each diet/period combination the most abundant phyla were the *Bacteroidetes* followed by the *Firmicutes* with a decrease in the quantity of *Bacteroidetes* and corresponding increase in the *Firmicutes* from time period 1 to 2 (**Figure 1** shows the relative abundance of each phylum found for all diet/ period combinations). When a phylogenetic tree was constructed (using the ‘hclust’ package in R with Bray Curtis dissimilarity) there was no grouping of samples according to diet, period, or any other factors known about the sample animals (**Figure 2**).

### Core Bacterial Community

**FIGURE 3:**
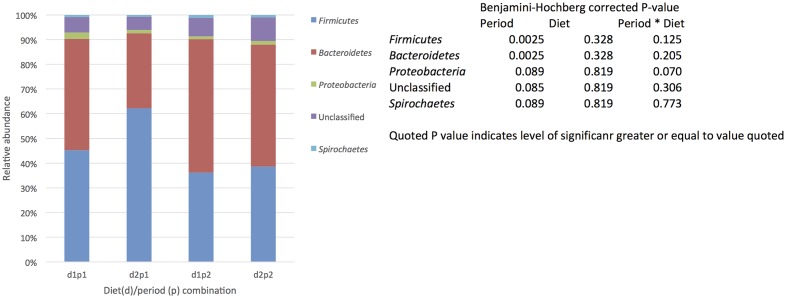
The effect of time (p: period 1 vs. period 2, 6 weeks apart) and diet (d: diet 1 = hay plus chaff diet, diet 2 = hay plus balancer) on the relative abundance (%) of bacterial phyla making up the core microbiome (defined by being present in all samples (each animal/diet combination) included in the study and being present at 0.1% relative abundance or greater,) in equine feces.

The presence of a core community defined by being present in all samples (each animal/diet combination) included in the study and being present at 0.1% relative abundance (or greater). was identified. In total 65 OTUs were identified that fit the definition of core making up 21–28% of the total sequences recovered (Supplementary Table S3). As with total population the most abundant phyla were the *Bacteroidetes* followed by the *Firmicutes*, however, there was no obvious shift in phyla due to period (**Figure 3**). Indeed when the relative abundance of OTUs was examined across diets and periods only a single OTU identified as *Streptococcus* was effected by diet and there were no other significant effect of diet or period alone or in combination on the relative abundance of the core OTUs (Supplementary Table S3).

### Stability

**FIGURE 4:**
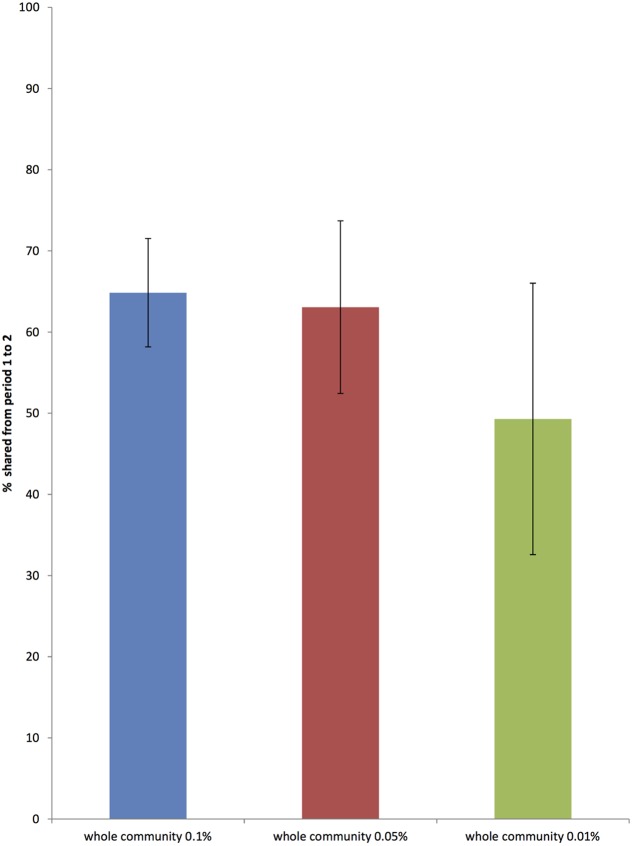
The proportion of the whole bacterial community that is common to sample periods 1 and 2, 6 weeks apart at three different levels of relative abundance (0.01, 0.05, and 0.1 %) (Error bars represent standard deviation across 12 horses).

The average stability of the total bacterial community between animals, as shown by the Jaccard index, was 0.64, *SD* = 0.05. **Figure 4** shows the stability of total bacterial communities at each described level of relative abundance. Supplementary Table S4 shows the Jaccard index across all 12 animals.

### Changes in Bacteria Over Time and With Diet

When bacterial OTUs were compared statistically between time points OTUs belonging to five genera (*Lactobacillus, Roseburia, Butyrivibrio, Streptococcus* and an unclassified genus belonging to the *Acidaminococcaceae* family) showed a significant increase from time period 1 to 2 and OTUs belonging to 6 genera (*Anaeroplasma, Anaerophaga, Phocaeicola*, and two unclassified genera belong to the *Firmicutes* and *Bacteroidetes* phyla and one totally unclassified genera) showed a significant decrease (see **Table 2**). When bacterial OTUs were compared statistically between diets OTUs belonging to five genera (*Lactobacillus, Campylobacter, Flavonifractor* and unclassified genera from the order *Sphingobacteriales* and class *Deltaproteobacteria)* showed a significant increase from diet 1 to diet 2 and OTUs belonging to 6 genera (*Robinsoniella, Clostridium sensu stricto, Lachnospiracea, Erysipelotrichaceae, Anaerophaga*, and an unclassified genera of the order *Bacillales)* showed a significant decrease (see **Table 3**), there were no significant interactions between time and diet.

**Table 2:** Bacterial operational taxonomic units (OTUs) significantly different between periods 1 and 2 (both diets 1 and 2 combined).

Phylum	Class	Order	Family	Genus	Corrected *p*-value	log2Fold change	Average relative abundance Period 1	*SD*	Average relative abundance Period 2	*SD*
*Tenericutes*	*Mollicutes*	*Anaeroplasmatales*	*Anaeroplasmataceae*	*Anaeroplasma*	0.035	-2.06	0.05	0.05	0.14	0.12
*Firmicutes*	Unclassified	Unclassified	Unclassified	Unclassified	0.035	-1.21	1.17	0.51	1.80	1.11
*Unclassified*	Unclassified	Unclassified	Unclassified	Unclassified	0.035	-0.98	4.98	1.60	6.56	2.06
*Bacteroidetes*	*Bacteroidia*	*Bacteroidales*	Unclassified	Unclassified	0.035	-0.72	18.52	3.48	24.56	5.15
*Firmicutes*	*Bacilli*	*Lactobacillales*	*Lactobacillaceae*	*Lactobacillus*	0.035	1.76	0.80	0.88	0.24	0.21
*Firmicutes*	*Clostridia*	*Clostridiales*	*Lachnospiraceae*	*Roseburia*	0.035	1.82	0.08	0.05	0.04	0.04
*Firmicutes*	*Clostridia*	*Clostridiales*	*Lachnospiraceae*	*Butyrivibrio*	0.071	1.60	0.13	0.09	0.09	0.10
*Firmicutes*	*Bacilli*	*Lactobacillales*	*Streptococcaceae*	*Streptococcus*	0.073	1.65	7.60	4.84	2.82	2.29
*Bacteroidetes*	*Bacteroidia*	*Bacteroidales*	*Marinilabiaceae*	*Anaerophaga*	0.096	-2.74	0.62	1.50	1.26	1.90
*Bacteroidetes*	*Bacteroidia*	*Bacteroidales*	*Bacteroidales incertae sedis*	*Phocaeicola*	0.096	-1.93	0.58	0.40	1.62	2.38
*Firmicutes*	*Negativicutes*	*Selenomonadales*	*Acidaminococcaceae*	Unclassified	0.096	1.20	0.16	0.10	0.13	0.10

*A positive log2 fold change relates to a decrease in relative abundance from period 1 to 2, a negative log2 fold relates to an increase. SD = standard deviation*.

**Table 3:** Bacterial OTUs significantly different between diets 1 (hay plus chaff) and 2 (hay plus balancer) (both periods 1 and 2 combined).

Phylum	Class	Order	Family	Genus	Corrected P-value	log2Fold change	Average relative abundance Diet 1	*SD*	Average relative abundance Diet 2	*SD*
*Firmicutes*	*Bacilli*	*Lactobacillales*	*Lactobacillaceae*	*Lactobacillus*	0.001	2.70	0.68	0.49	0.20	0.18
*Firmicutes*	*Clostridia*	*Clostridiales*	*Lachnospiraceae*	*Robinsoniella*	0.012	-2.82	0.00	0.00	0.04	0.03
*Proteobacteria*	*Epsilonproteobacteria*	*Campylobacterales*	*Campylobacteraceae*	*Campylobacter*	0.012	4.66	0.02	0.02	0.00	0.00
*Firmicutes*	*Clostridia*	*Clostridiales*	*Clostridiaceae 1*	*Clostridium sensu stricto*	0.016	-3.06	0.01	0.01	0.08	0.12
*Firmicutes*	*Clostridia*	*Clostridiales*	*Lachnospiraceae*	*Lachnospiracea*	0.048	-0.89	0.78	0.32	1.93	0.63
*Proteobacteria*	*Deltaproteobacteria*	Unclassified	Unclassified	Unclassified	0.053	3.37	0.05	0.08	0.03	0.05
*Firmicutes*	*Erysipelotrichia*	*Erysipelotrichales*	*Erysipelotrichaceae*	*Erysipelotrichaceae*	0.058	-1.47	0.09	0.07	0.34	0.17
*Firmicutes*	*Bacilli*	*Bacillales*	Unclassified	Unclassified	0.070	-3.38	0.00	0.01	0.02	0.01
*Bacteroidetes*	*Bacteroidia*	*Bacteroidales*	*Marinilabiaceae*	*Anaerophaga*	0.071	-2.85	0.61	1.37	1.44	1.92
*Firmicutes*	*Clostridia*	*Clostridiales*	*Ruminococcaceae*	*Flavonifractor*	0.089	1.70	0.27	0.17	0.10	0.11
*Bacteroidetes*	*Sphingobacteria*	*Sphingobacteriales*	Unclassified	Unclassified	0.098	1.25	2.21	1.37	1.34	0.80

*A positive log2 fold change relates to a decrease in relative abundance from diets 1 to 2, a negative log2 fold relates to an increase. SD = standard deviation*.

## Discussion

The bacterial community found in the gastrointestinal tract of mammals is shaped by many factors. After initial colonization at birth (Wang et al., 2009; Song et al., 2013), the bacterial community develops and changes until the animal reaches adulthood. Diet appears to have the biggest influence in driving the community structure with the intestinal microbiome of omnivores, carnivores and herbivores shown to be pylogenetically distinct from each other (Ley et al., 2008). Temporal stability of the community has begun to be investigated to understand if patterns described during single time point studies are maintained over time. Once this is understood, more accurate comparisons can be made to disease states and diagnostic tools can be developed (Aziz et al., 2013; Lozupone et al., 2013). It has been suggested that intestinal microbiome stability during adulthood may be a feature of mammals (Faith et al., 2013; Becker et al., 2015)

In this study, microbial samples from two time periods (6 weeks apart) from individual horses phylogenetically clustered by horse showing a high degree of inter animal variability. This is consistent with other work in the horse where samples have been collected over time (Willing et al., 2009; Blackmore et al., 2013) and in single time point studies where again large inter-horse variability has been shown (Costa et al., 2012; Steelman et al., 2012). Sample replicates taken over 12 h during both time periods clustered tightly together by horse, with no grouping according to diet. This is again consistent with findings of Blackmore et al. (2013), where samples collected over 72 h grouped closely together for each animal. This grouping was also found with sample replicates taken over 1 day in our previous work (Dougal et al., 2014). The largest discriminating factor relating to the bacterial community of the hindgut of the horse seems to be that of animal to animal variability. The differences found in this study compared to other studies may be explained by the higher resolution obtained through the use of 454 pyrosequencing compared to TRFLP. In the current study, there appears to be a higher degree of temporal intra animal stability than previously shown. However, such a community wide comparison does not consider which community members remain stable over time and the relative importance of those which do.

The most abundant phyla found were the *Bacteroidetes* (52%) and *Firmicutes* (35%), with smaller quantities (<4%) of *Spirochaetes, Fibrobacteres, Proteobacteria*, and *Actinobacteria*. The identification of *Bacteroidetes* and *Firmicutes* as the most abundant phyla is consistent with our previous findings and published values for other mammalian species (Eckburg et al., 2005; Gill et al., 2006; Backhed et al., 2005). However, values previously reported for *Bacteroidetes* using next generation sequencing approaches in the horse are generally lower (Shepherd et al., 2012; Costa et al., 2012; Steelman et al., 2012). The values reported here are, however, only marginally higher than values reported (45–49%) in older, culture independent studies (Yamano et al., 2008; Willing et al., 2009) and in a recent 454 pyrosequencing study by Proudman et al. (2015), reporting a similar abundance (42%). In comparison to our previous work, in this study the abundance of *Bacteroidetes* was higher and there was a clear reduction in this phylum with a concordant increase in *Firmicutes* from period 1 to time 2. Changes in intestinal microbiota associated with dietary restriction in man have been reviewed recently but contrary to the results found here, restrictive diets in man generally decreased the microbiota abundance and reduced *Firmicutes* numbers (Seganfredo et al., 2017).

The samples obtained for this study were from clinically obese horses, which underwent a weight reduction program through reduced calorie intake. In humans it is known that intestinal microbiome biodiversity is significantly lower in obese individuals (Turnbaugh et al., 2009), however, we saw no significant effects in diversity between periods in this experiment. There have been reports in humans of changes in the quantity of the *Bacteroidetes* and *Firmicutes* phyla associated with the obese state (Turnbaugh et al., 2006; Duncan et al., 2008; Martinez et al., 2013). Most publications in humans report a decrease in *Bacteroidetes* in obese individuals with a compensatory increase in *Firmicutes*, when compared to non-obese subjects (Ley et al., 2006; Turnbaugh et al., 2006; Martinez et al., 2013). However, some publications report either no difference (Duncan et al., 2008; Zhang et al., 2009) or an increased abundance of *Bacteroidetes* (Schwiertz et al., 2010) in obese compared to normal body weight individuals. Furthermore, it is important to consider differences found within families/ genus of bacteria, as differences at the phylum level may be misleading (Zhang et al., 2009). For example, within the *Bacteroidetes* phyla an enrichment specifically of the *Prevotella* family has been found, whilst *Erysiplotrichaceae* (*Firmicutes*) were also enriched in obese individuals (Zhang et al., 2009). Findings of our previous work relating to diet and age effects on the bacterial community also highlight this issue, as some OTUs belonging to the same bacterial family increase whilst some decrease in response to treatment (Dougal et al., 2014). It is difficult to comment on the significance of the differences seen at phylum level (in this study), other than to highlight that the bacterial community does undergo change in response to being obese and through weight loss. Thus, whilst it is tempting to try to link the changes in microbiome to live weight and specifically weight loss in individual animals (Argo et al., 2012), the number of animals per diet is small (5) and as such we believe it is inappropriate to relate the microbiome to the animal phenotype.

The most abundant members of the core community identified in this study were *Bacteroidetes* and *Firmicutes* at phylum level and *Streptococcaceae, Prevotellaceae*, *Porphyromonadaceae Lachnospiraceae*, and an unclassified phylotype at the family level. The size of the fecal core bacterial community identified in this study (65 OTUs making up 21–28% of the total sequences recovered) was somewhat larger than what we have previously shown (25 OTUs, 14% (Dougal et al., 2013) and 30 OTUs, 16% for fiber based diet (Dougal et al., 2014). However, this is still considerably smaller than the core identified in other species (Qin et al., 2010; Turnbaugh et al., 2010; Sekelja et al., 2011; Jami and Mizrahi, 2012). With the exception of the *Streptococcaceae* family these families have been previously identified as part of the core community in the horse when fed a high fiber diet (Dougal et al., 2014). One notable family missing from the core identified in this study in comparison to our earlier work is the family *Fibrobacteriaceae*, suggesting a potential link with its absence and obesity or weight loss. However, further investigation must be carried out before commenting further. Similarly, the identification of the family *Streptococcaceae* as part of the core community in this chapter is a potentially interesting finding due to this family not appearing as part of a core community in our previous work or indeed in the human literature (Turnbaugh et al., 2010, Dougal et al., 2013, 2014). Work by Steelman et al. (2012), however, identified *Streptococcaceae* as the most abundant family of bacteria in the horses included in their work.

When considering how stable the bacterial community in the hindgut of the horse is we have shown that over a 6-week period (with animals on a uniform diet) 65% of bacteria are retained. This shows a reasonable degree of stability in the short-term, although lower than that recently demonstrated for humans. Work by Faith et al. (2013) shows humans retain greater than 70% of bacteria after a one year period and still retain around 60% after 5 years.

Temporal stability in the members of the core bacterial community has, to the author’s knowledge, not been investigated in the horse. This question has received limited attention in humans but the core population has been shown to be stable over time (Jalanka-Tuovinen et al., 2011; Sekelja et al., 2011) and indeed Sekelja et al. (2011) suggested that for a core community to be truly core it must be stable over time. Indeed, it is essential to characterize the core community that is stable over time, in healthy animals on a fiber based diet, to enable comparisons to be made with different diets and disease states. Here it has been shown that a core community does persist over time (6 weeks) in horses on a fiber based diet with relatively minor changes in the relative abundance of different species. The most abundant members of the core that are maintained over this time are the same families that we have previously identified (Dougal et al., 2013, 2014) as accounting for the largest part of the core population. Recent work in humans has suggested that bacteria belonging to some phyla are more stable over time than others; *Bacteroidetes* and *Actinobacteria* more so than *Firmicutes* and *Proteobacteria* (Faith et al., 2013). It has also been suggested that in humans the core bacterial community has greater stability in individuals than the non-core population (Jalanka-Tuovinen et al., 2011; Sekelja et al., 2011). This suggests that the core may be more resilient to challenge, allowing the host to be unaffected by minor perturbation. Indeed, when Sekelja et al. (2011) considered the data from Dethlefsen et al. (2008) antibiotic treatment was shown to have little effect on the core phylotypes.

## Conclusion

Understanding how the bacterial community of the horse changes (or does not) over time is essential to provide a baseline for interpretation of dietary management or disease impacts. In this study, we show that there is a degree of stability, 65% of bacteria are retained, within the bacterial community of the hindgut of horses over a 6 week period whilst on a uniform diet. This degree of stability is not markedly lower than values reported for the human gut. While this work explored stability of the bacterial community at the end of a weight loss trial, when body weight was stable, further work is needed to look at changes in the microbiota during the weight loss phase.

## Author Contributions

Conception and design of original study where samples were obtained (PH, GC, CB, CA), Conception and design of microbiological study (KD, PH, CN). Acquisition of data (KD, SG). Analysis of data (KD, SG, CC, CN). Interpretation of data (KD, PH, CC, CN). Drafting of work (KD, PH, CC, CA, CN). Revising work critically (KD, PH, SG, CC, GC, CB, CN). Final approval of version to be published (KD, PH, SG, CC, GC, CB, CA). Agreement to be accountable for all aspects of the work (KD, PH, SG, CC, GC, CB, CA, CN).

## Conflict of Interest Statement

PH and CB are employed by one of the funders of this research (WALTHAM Centre for Pet Nutrition, Melton Mowbray, Leicestershire. LE14 4RT). The authors can confirm that they have adhered to all the required policies on sharing data and materials. The other authors declare that the research was conducted in the absence of any commercial or financial relationships that could be construed as a potential conflict of interest.

## Footnotes

1 http://weizhongli-lab.org/cd-hit-otu/

2 http://www.festinalente.me/bioinf/

3 www.r-project.org/
