# Supplementary material for: Changes in the Total Fecal Bacterial Population in Individual Horses Maintained on a Restricted Diet Over 6 Weeks
Source: Front Microbiol. 2017 Aug 11;8:1502. doi: 10.3389/fmicb.2017.01502 (PMC5554519; doi:10.3389/fmicb.2017.01502)
Supplement: Supplementary file 1 [file Table_1.DOCX]

***Table S1-*** *Animal metadata from Liverpool weight loss study*

| Animal number | Breed | Age (years) | Sex | BM (Kg) | BCS* | Diet** |
| --- | --- | --- | --- | --- | --- | --- |
| 1 | Shetland pony | 9 | Gelding | 233 | 8.3 | 1 |
| 2 | Thoroughbred cross | 5 | Mare | 358 | 8.0 | 1 |
| 3 | Cob | 12 | Gelding | 478 | 8.2 | 1 |
| 4 | Cob | 13 | Mare | 501 | 7.0 | 1 |
| 5 | Cob | 11 | Mare | 598 | 7.0 | 1 |
| 6 | Warmblood | 10 | Gelding | 764 | 7.3 | 1 |
| 7 | Welsh cob x Thoroughbred | 16 | Mare | 638 | 8.5 | 2 |
| 8 | Cob | 16 | Mare | 606 | 8.3 | 2 |
| 9 | Cob | 13 | Gelding | 600 | 8.5 | 2 |
| 10 | Cob | 5 | Gelding | 556 | 8.3 | 2 |
| 11 | Welsh pony | 14 | Mare | 277 | 8.2 | 2 |
| 12 | Shetland pony | 12 | Gelding | 195 | 7.0 | 2 |

*BCS-Body condition score

**(1-Hay +chaff diet ;2- Hay +balancer)
