# Supplementary material for: Changes in the Total Fecal Bacterial Population in Individual Horses Maintained on a Restricted Diet Over 6 Weeks
Source: Front Microbiol. 2017 Aug 11;8:1502. doi: 10.3389/fmicb.2017.01502 (PMC5554519; doi:10.3389/fmicb.2017.01502)
Supplement: Supplementary file 2 [file Table_2.DOCX]

**Table S2** Diet composition (Diet 1=hay plus chaff diet, Diet 2=hay plus balancer)

|  | **Hay** | **Chaff diet** | **Balancer** |
| --- | --- | --- | --- |
| **Dry Matter**  **(%)** | 88.40 | 88.14 | 88.42 |
| **Gross Energy**  **(MJ/kg DM)** | 19.97 | 19.79 | 18.76 |
| **Crude Protein**  **(%)** | 7 | 12 | - |
| **Acid detergent fibre (MJ/kg DM)** | 431 | 387 | 79 |
| **Ash**  **(%)** | 3.80 | _ | _ |
