# Supplementary material for: Changes in the Total Fecal Bacterial Population in Individual Horses Maintained on a Restricted Diet Over 6 Weeks
Source: Front Microbiol. 2017 Aug 11;8:1502. doi: 10.3389/fmicb.2017.01502 (PMC5554519; doi:10.3389/fmicb.2017.01502)
Supplement: Supplementary file 3 [file Table_3.PDF]

Table S3. The relative abundance of OTUs identified as being part of the core microbiome (defined by being present in all samples (each animal/diet combination) included in the study and being present at 0.1% relative abundance or greater) , over time (period 1 v period 2, 6 weeks apart) and diet (Diet 1=hay plus chaff diet, Diet 2=hay plus balancer)

OTU	Phylum	Class	Order	Family	Genus	Relative abundance				SED			Benjamini-Hochberg corrected P-value		
						Period 1		Period 2							
						Diet 1	Diet 2	Diet 1	Diet 2	Period	Diet	Period * Diet	Period	Diet	Period * Diet
1	*Firmicutes*	*Bacilli*	*Lactobacillales*	*Streptococcaceae*	*Streptococcus*	4.25	9.34	1.40	2.43	0.870	1.248	1.521	0.442	0.065	0.775
4	*Bacteroidetes*	*Bacteroidia*	*Bacteroidales*	Unclassified	Unclassified	1.67	0.77	1.30	0.96	0.522	0.780	0.939	0.856	0.915	0.866
10	*Bacteroidetes*	*Bacteroidia*	*Bacteroidales*	*Prevotellaceae*	*Paraprevotella*	1.53	1.42	0.98	1.56	0.286	0.465	0.546	0.880	0.682	0.808
21	*Bacteroidetes*	*Bacteroidia*	*Bacteroidales*	*Porphyromonadaceae*	Unclassified	1.20	0.31	1.70	0.22	0.489	0.426	0.648	0.442	0.823	0.838
22	*Firmicutes*	Unclassified	Unclassified	Unclassified	Unclassified	0.28	0.31	0.95	0.72	0.302	0.422	0.519	0.920	0.372	0.676
24	*Firmicutes*	*Bacilli*	*Lactobacillales*	*Lactobacillaceae*	*Lactobacillus*	0.31	1.14	0.10	0.25	0.201	0.194	0.279	0.442	0.181	0.775
32	*Bacteroidetes*	*Bacteroidia*	*Bacteroidales*	Unclassified	Unclassified	0.42	0.69	0.56	1.14	0.148	0.326	0.357	0.703	0.301	0.808
33	*Bacteroidetes*	*Bacteroidia*	*Bacteroidales*	*Porphyromonadaceae*	*Barnesiella*	0.31	0.18	0.39	0.28	0.063	0.867	0.107	0.701	0.503	1.005
40	*Bacteroidetes*	*Bacteroidia*	*Bacteroidales*	Unclassified	Unclassified	0.26	0.21	0.31	0.23	0.046	0.157	0.163	0.880	0.682	0.932
43	*Bacteroidetes*	*Bacteroidia*	*Bacteroidales*	*Prevotellaceae*	Unclassified	0.80	0.38	0.42	0.41	0.263	0.277	0.382	0.856	0.682	0.827
49	*Firmicutes*	*Clostridia*	*Clostridiales*	*Lachnospiraceae*	Unclassified	0.16	0.17	0.18	0.21	0.029	0.100	0.104	0.920	0.580	0.931
50	*Bacteroidetes*	*Bacteroidia*	*Bacteroidales*	*Porphyromonadaceae*	Unclassified	0.34	0.41	0.35	0.57	0.049	0.171	0.178	0.856	0.374	0.775
52	*Firmicutes*	*Clostridia*	*Clostridiales*	*Lachnospiraceae*	Unclassified	0.79	0.65	0.94	0.46	0.110	0.269	0.291	0.755	0.915	0.808
53	*Bacteroidetes*	*Bacteroidia*	*Bacteroidales*	*Porphyromonadaceae*	*Barnesiella*	0.82	0.96	0.47	0.59	0.314	0.267	0.412	0.880	0.580	1.006
63	Unclassified	Unclassified	Unclassified	Unclassified	Unclassified	0.33	0.82	0.25	0.76	0.161	0.191	0.249	0.442	0.820	1.006
64	*Firmicutes*	*Clostridia*	*Clostridiales*	*Lachnospiraceae*	Unclassified	0.47	0.27	0.18	0.26	0.075	0.108	0.131	0.880	0.301	0.775
65	*Firmicutes*	*Clostridia*	*Clostridiales*	*Ruminococcaceae*	*Oscillibacter*	0.61	0.15	0.61	0.17	0.115	0.144	0.184	0.442	0.960	1.000
68	*Bacteroidetes*	*Bacteroidia*	*Bacteroidales*	*Porphyromonadaceae*	Unclassified	0.40	0.20	0.36	0.21	0.060	0.143	0.155	0.733	0.915	0.932
71	*Firmicutes*	*Clostridia*	*Clostridiales*	*Lachnospiraceae*	Unclassified	0.34	0.17	0.36	0.14	0.061	0.127	0.140	0.654	0.960	0.932
78	*Bacteroidetes*	Unclassified	Unclassified	Unclassified	Unclassified	0.12	0.14	0.15	0.14	0.018	0.038	0.042	1.000	0.730	0.832
84	*Bacteroidetes*	*Bacteroidia*	*Bacteroidales*	*Prevotellaceae*	Unclassified	0.19	0.22	0.19	0.28	0.038	0.095	0.102	0.880	0.682	0.813
95	*Firmicutes*	*Clostridia*	*Clostridiales*	*Lachnospiraceae*	Unclassified	0.09	0.29	0.07	0.17	0.044	0.074	0.086	0.473	0.421	0.808
103	*Firmicutes*	*Clostridia*	*Clostridiales*	*Lachnospiraceae*	Unclassified	0.45	0.79	0.46	0.55	0.107	0.167	0.198	0.703	0.580	0.808
105	Unclassified	Unclassified	Unclassified	Unclassified	Unclassified	0.27	0.24	0.31	0.30	0.055	0.104	0.117	0.921	0.660	1.005
106	Unclassified	Unclassified	Unclassified	Unclassified	Unclassified	0.18	0.04	0.25	0.12	0.059	0.117	0.131	0.755	0.546	1.006
111	*Bacteroidetes*	*Bacteroidia*	*Bacteroidales*	*Prevotellaceae*	*Hallella*	0.16	0.11	0.13	0.10	0.020	0.039	0.044	0.755	0.618	0.827
114	*Bacteroidetes*	Unclassified	Unclassified	Unclassified	Unclassified	0.20	0.11	0.20	0.23	0.047	0.063	0.078	0.880	0.556	0.808
118	Unclassified	Unclassified	Unclassified	Unclassified	Unclassified	0.20	0.19	0.30	0.42	0.062	0.116	0.131	0.880	0.181	0.808
122	*Spirochaetes*	*Spirochaetes*	*Spirochaetales*	Unclassified	Unclassified	0.21	0.23	0.24	0.25	0.036	0.154	0.158	0.995	0.682	1.005
125	*Bacteroidetes*	*Bacteroidia*	*Bacteroidales*	*Prevotellaceae*	*Paraprevotella*	0.25	0.32	0.42	0.44	0.101	0.130	0.165	0.920	0.512	1.005
127	*Firmicutes*	*Negativicutes*	*Selenomonadales*	*Veillonellaceae*	Unclassified	0.06	0.42	0.15	0.17	0.105	0.103	0.147	0.498	0.682	0.775
128	*Bacteroidetes*	*Bacteroidia*	*Bacteroidales*	*Porphyromonadaceae*	*Barnesiella*	0.15	0.15	0.17	0.10	0.049	0.050	0.070	0.880	0.854	0.834
130	*Firmicutes*	*Clostridia*	*Clostridiales*	*Lachnospiraceae*	*Lachnospiracea_incertae_sedis*	0.49	0.34	0.19	0.24	0.081	0.142	0.163	0.920	0.206	0.808
138	*Bacteroidetes*	*Bacteroidia*	*Bacteroidales*	*Rikenellaceae*	*Rikenella*	0.09	0.11	0.14	0.16	0.024	0.026	0.035	0.880	0.301	1.006
142	*Firmicutes*	*Clostridia*	*Clostridiales*	*Lachnospiraceae*	Unclassified	0.22	0.13	0.29	0.19	0.063	0.112	0.129	0.856	0.580	1.006
146	*Bacteroidetes*	*Bacteroidia*	*Bacteroidales*	Unclassified	Unclassified	0.84	0.22	0.43	0.37	0.162	0.189	0.249	0.520	0.682	0.775
147	*Firmicutes*	*Clostridia*	*Clostridiales*	*Lachnospiraceae*	Unclassified	0.13	0.11	0.09	0.04	0.021	0.020	0.029	0.473	0.206	0.808
162	*Firmicutes*	*Clostridia*	*Clostridiales*	*Ruminococcaceae*	Unclassified	0.06	0.25	0.23	0.13	0.082	0.089	0.121	0.880	0.907	0.775
166	*Firmicutes*	*Clostridia*	*Clostridiales*	*Lachnospiraceae*	*Cellulosilyticum*	0.23	0.50	0.06	0.28	0.051	0.117	0.127	0.473	0.098	0.589
190	*Bacteroidetes*	Unclassified	Unclassified	Unclassified	Unclassified	0.08	0.17	0.12	0.13	0.044	0.051	0.067	0.782	0.944	0.808
194	*Bacteroidetes*	*Bacteroidia*	*Bacteroidales*	*Rikenellaceae*	*Rikenella*	0.18	0.05	0.18	0.11	0.030	0.063	0.070	0.654	0.613	0.808
196	*Firmicutes*	*Clostridia*	*Clostridiales*	*Ruminococcaceae*	Unclassified	0.09	0.24	0.04	0.17	0.073	0.071	0.102	0.473	0.682	1.005
197	*Firmicutes*	*Clostridia*	*Clostridiales*	*Ruminococcaceae*	Unclassified	0.19	0.19	0.16	0.19	0.033	0.048	0.058	0.920	0.820	1.005
199	*Firmicutes*	*Clostridia*	*Clostridiales*	*Ruminococcaceae*	Unclassified	0.14	0.23	0.05	0.07	0.035	0.051	0.062	0.755	0.098	0.808
210	*Bacteroidetes*	*Bacteroidia*	*Bacteroidales*	Unclassified	Unclassified	0.14	0.08	0.16	0.22	0.028	0.032	0.043	1.000	0.181	0.775
215	*Firmicutes*	*Clostridia*	*Clostridiales*	*Lachnospiraceae*	*Coprococcus*	0.09	0.15	0.09	0.09	0.018	0.028	0.031	0.782	0.374	0.775
226	*Proteobacteria*	Unclassified	Unclassified	Unclassified	Unclassified	0.52	0.34	0.23	0.30	0.052	0.093	0.106	0.880	0.130	0.775
227	Unclassified	Unclassified	Unclassified	Unclassified	Unclassified	0.18	0.10	0.19	0.10	0.021	0.040	0.045	0.473	0.915	0.838
250	*Bacteroidetes*	*Bacteroidia*	*Bacteroidales*	Unclassified	Unclassified	0.18	0.11	0.15	0.23	0.058	0.050	0.076	0.992	0.682	0.808
259	*Bacteroidetes*	*Bacteroidia*	*Bacteroidales*	Unclassified	Unclassified	0.08	0.10	0.53	0.30	0.131	0.162	0.208	0.880	0.206	0.808

261	*Bacteroidetes*	*Bacteroidia*	*Bacteroidales*	Unclassified	Unclassified	0.05	0.27	0.46	0.33	0.198	0.192	0.275	0.920	0.579	0.808
270	*Bacteroidetes*	*Bacteroidia*	*Bacteroidales*	Unclassified	Unclassified	0.09	0.12	0.22	0.15	0.059	0.073	0.093	0.920	0.512	0.808
284	*Bacteroidetes*	*Bacteroidia*	*Bacteroidales*	Unclassified	Unclassified	0.23	0.08	0.28	0.24	0.082	0.105	0.134	0.856	0.546	0.832
321	*Firmicutes*	*Negativicutes*	*Selenomonadales*	*Veillonellaceae*	Unclassified	0.22	0.51	0.13	0.29	0.098	0.160	0.188	0.701	0.428	0.832
323	*Bacteroidetes*	*Bacteroidia*	*Bacteroidales*	*Prevotellaceae*	*Prevotella*	0.08	0.10	0.16	0.08	0.023	0.047	0.052	0.856	0.556	0.775
327	*Firmicutes*	*Clostridia*	*Clostridiales*	*Lachnospiraceae*	Unclassified	0.15	0.12	0.17	0.20	0.043	0.068	0.080	1.000	0.580	0.832
328	*Bacteroidetes*	*Bacteroidia*	*Bacteroidales*	Unclassified	Unclassified	0.09	0.31	0.61	0.28	0.224	0.245	0.332	0.920	0.580	0.808
332	*Firmicutes*	*Clostridia*	*Clostridiales*	*Lachnospiraceae*	Unclassified	0.14	0.13	0.03	0.08	0.030	0.044	0.053	0.880	0.181	0.808
344	*Firmicutes*	*Clostridia*	*Clostridiales*	*Ruminococcaceae*	*Ruminococcus*	0.10	0.25	0.13	0.19	0.042	0.046	0.062	0.473	0.854	0.808
359	Unclassified	Unclassified	Unclassified	Unclassified	Unclassified	0.12	0.08	0.22	0.14	0.042	0.055	0.069	0.782	0.348	0.808
371	*Firmicutes*	*Clostridia*	*Clostridiales*	*Lachnospiraceae*	*Cellulosilyticum*	0.19	0.21	0.08	0.13	0.026	0.074	0.079	0.880	0.098	0.837
415	*Firmicutes*	*Clostridia*	*Clostridiales*	*Ruminococcaceae*	*Oscillibacter*	0.13	0.11	0.11	0.10	0.029	0.053	0.060	0.920	0.730	1.014
470	*Firmicutes*	*Clostridia*	*Clostridiales*	*Lachnospiraceae*	Unclassified	0.11	0.07	0.09	0.09	0.022	0.042	0.048	0.880	0.978	0.813
538	*Bacteroidetes*	*Bacteroidia*	*Bacteroidales*	Unclassified	Unclassified	0.11	0.18	0.12	0.31	0.036	0.091	0.098	0.701	0.301	0.775
561	*Firmicutes*	*Negativicutes*	*Selenomonadales*	*Acidaminococcaceae*	*Succinispira*	0.14	0.12	0.13	0.09	0.022	0.025	0.033	0.701	0.660	0.866

Corrected P-values were considered significant <0.1
