# Supplementary material for: Changes in the Total Fecal Bacterial Population in Individual Horses Maintained on a Restricted Diet Over 6 Weeks
Source: Front Microbiol. 2017 Aug 11;8:1502. doi: 10.3389/fmicb.2017.01502 (PMC5554519; doi:10.3389/fmicb.2017.01502)
Supplement: Supplementary file 4 [file Table_4.DOCX]

***Table S4*** *Jaccard index*

| 1 | 2 | 3 | 4 | 5 | 6 | 7 | 8 | 9 | 10 | 11 | 12 | mean | st dev* |
| --- | --- | --- | --- | --- | --- | --- | --- | --- | --- | --- | --- | --- | --- |
| 0.67 | 0.63 | 0.65 | 0.62 | 0.74 | 0.62 | 0.68 | 0.62 | 0.57 | 0.63 | 0.57 | 0.62 | 0.64 | 0.05 |

*St dev= standard deviation
