# Supplementary material for: Changes in the Total Fecal Bacterial Population in Individual Horses Maintained on a Restricted Diet Over 6 Weeks
Source: Front Microbiol. 2017 Aug 11;8:1502. doi: 10.3389/fmicb.2017.01502 (PMC5554519; doi:10.3389/fmicb.2017.01502)
Supplement: Supplementary file 5 [file Image_1.pdf]

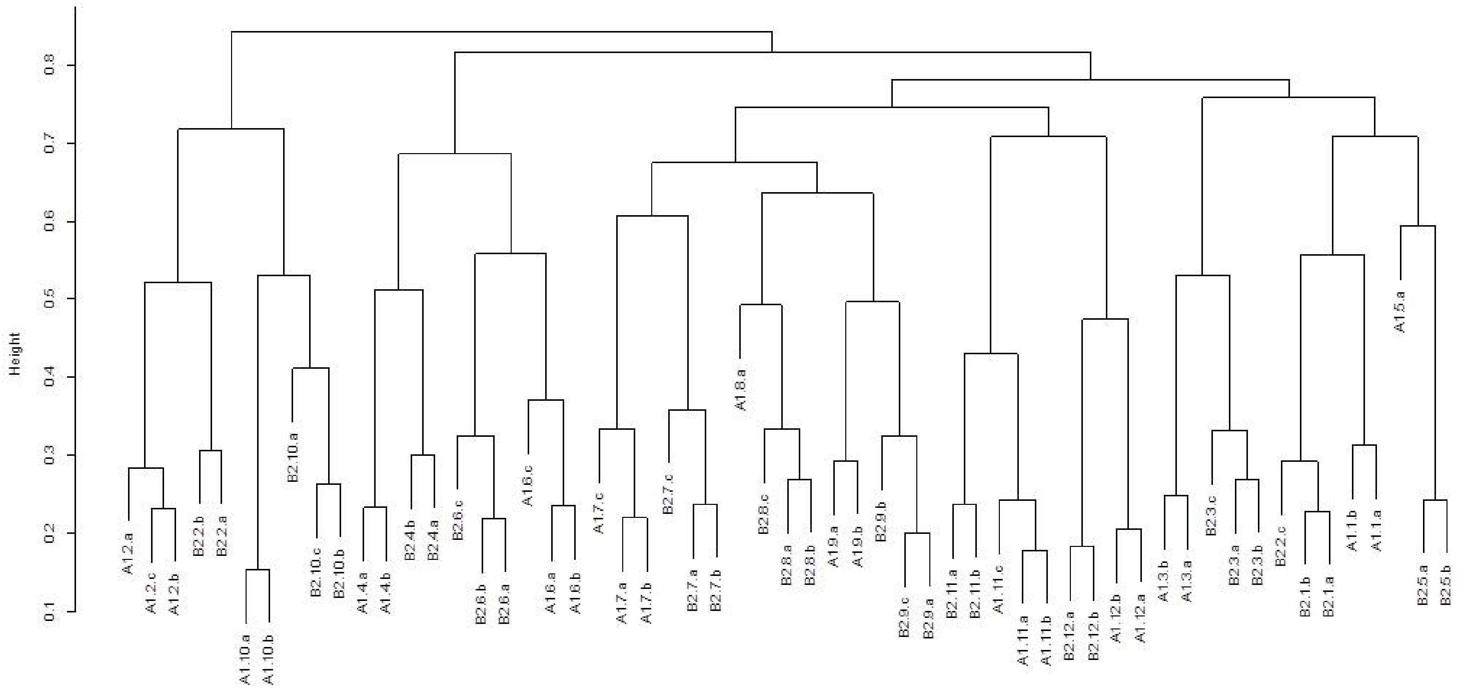

Figure S1 *Phylogenetic tree showing relationship between complete sample set (A1/B2 relates to sample day 1 and 2 respectively, 1-12 relates to horse number and a, b or c relates to sample replicate across sample day)*
