# Supplementary figures and images for: Changes in the Total Fecal Bacterial Population in Individual Horses Maintained on a Restricted Diet Over 6 Weeks
Source: Front Microbiol. 2017 Aug 11;8:1502. doi: 10.3389/fmicb.2017.01502 (PMC5554519; doi:10.3389/fmicb.2017.01502)

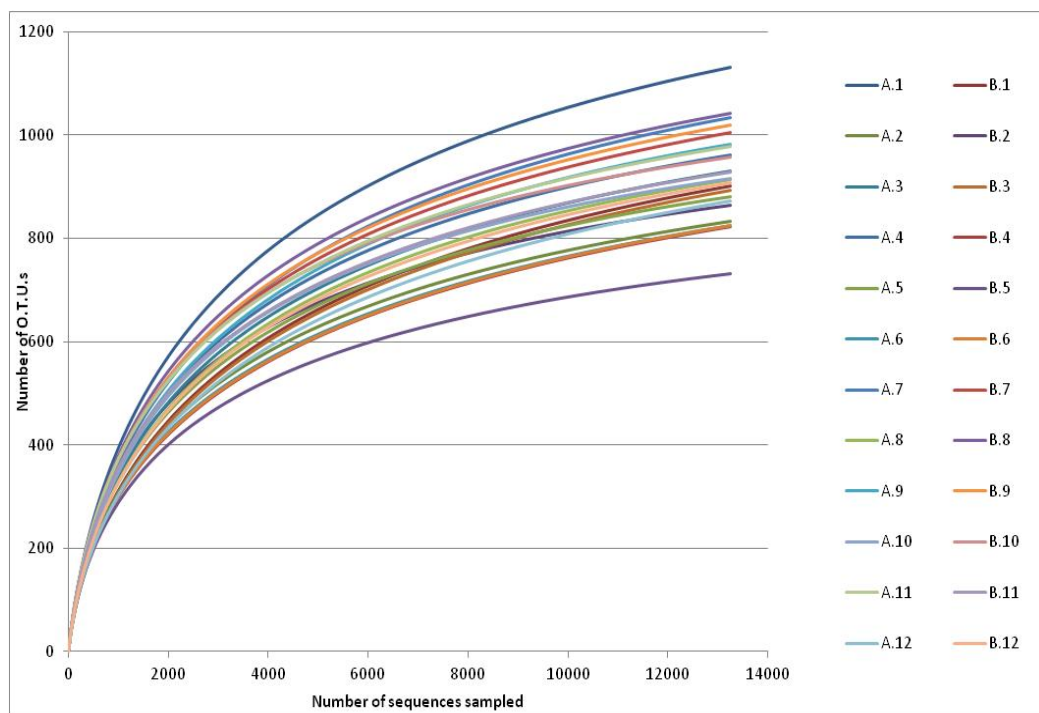

**Figure S2** *Rarefaction curves for samples after sample day replicates were combined*

Supplement: Supplementary file 6 [file Image_2.pdf]
